# Study of the Mechanism Underlying the Antihypertensive Effects of *Eucommia ulmoides* and *Tribulus terrestris* Based on an Analysis of the Intestinal Microbiota and Metabonomics

**DOI:** 10.1155/2020/4261485

**Published:** 2020-11-05

**Authors:** Ying-Zi Qi, Xue-Song Yang, Yue-Hua Jiang, Lin-Lin Shao, Ling-Yu Jiang, Chuan-Hua Yang

**Affiliations:** ^1^Health College, Shandong University of Traditional Chinese Medicine, Jinan 250355, China; ^2^Affiliated Hospital of Shandong University of Traditional Chinese Medicine, Jinan 250011, China; ^3^Traditional Chinese Medicine College, Tianjin University of Traditional Chinese Medicine, Tianjin 300000, China

## Abstract

The combination of *Eucommia ulmoides* and *Tribulus terrestris* (ET) has been widely utilized in clinical practice for thousands of years, but the mechanism underlying its efficacy has not been elucidated to date. This study attempted to investigate the role played by the intestinal microbiota and fecal metabolism in the response of elderly spontaneous hypertensive rats (SHRs) to ET administration as a treatment for hypertension. Fourteen male spontaneously hypertensive rats (SHRs, 18 months old) were randomly divided into an ET group and an SHR group, and 7 Wistar-Kyoto (WKY) rats of the same age were employed as the control group. The ET group was intragastrically administered 1.0 g/kg/d ET for 42 days, and SHRs and WKY rats were administered an equal amount of normal saline intragastrically. The intestinal microbiota and fecal metabolism were analyzed by 16S rRNA sequencing and the GC-MS (gas chromatography-mass spectrometry)/MS assay. ET treatment decreased blood pressure steadily, improved the colonic tissue morphology, and changed the structure and composition of the imbalanced microbiota in SHRs. Specifically, ET treatment increased the abundance of *Eubacterium*, which might be one of the target microbes for ET, and had a negative correlation with the levels of *α*-tocopherol, chenodeoxycholic acid, and deoxycholic acid according to the Spearman correlation analysis. The change in the intestinal microbiota affected the fecal metabolic pattern of SHRs. Eight potential biomarkers were determined to be primarily enriched in ABC transporters, phenylalanine metabolism, central carbon metabolism in cancer, purine metabolism, and protein digestion and absorption. The correlation analysis demonstrated that the abundance of *Eubacterium* and the decreased levels of *α*-tocopherol, chenodeoxycholic acid, and deoxycholic acid in the ET group were highly correlated. Our results suggest that ET has a good antihypertensive effect, which may be driven by the intestinal microbiota and their beneficial metabolites. The results of this study may help to elucidate the antihypertensive mechanism of ET.

## 1. Introduction

Accumulating evidence indicates that there is a close relationship between the intestinal microbiota and hypertension. The intestinal microbiome, which is considered the second human genome [1, 2], is believed to be closely related to chronic diseases, especially the occurrence and development of hypertension [3–6]. The overall balance in the composition of the intestinal microbiota is a key factor ensuring normal host functions [7, 8]. It has been suggested that the presence or absence of certain specific groups of intestinal microbiota contributes to immunity [9], nerve conduction [10], and energy supply [11]. Hypertension and imbalances of the intestinal microbiota influence each other, and the hypertensive phenotype can be “transferred” by fecal microbiota transplantation. The relationship between the intestinal microbiota and hypertension treatment merits further research.

*Eucommia ulmoides*, the bark of *Eucommia ulmoides Oliv.*, has the Chinese name Duzhong and the English name Eucommiae Cortex. The use of this bark has been recorded for thousands of years, and it is used to improve general health in traditional Chinese medicine. Pharmacological studies showed that aqueous and alcohol-based extracts of *Eucommia ulmoides* had the actions of lowering blood pressure and promoting bone-healing, antiaging, and vasodilatory effects. *Tribulus terrestris* has the Chinese name Cijili and the English name Puncturevine Caltrop Fruit. This plant grows widely in subtropical regions and is generally utilized to treat hypertension, arteriosclerosis, and aging. Our previous studies indicated that *Tribulus terrestris* decreases the generation of reactive oxygen species while increasing the concentration of NOS and improving the metabolism of endothelial cells [12]. The combination of *Eucommia ulmoides* and *Tribulus terrestris* (ET) has been widely employed in clinical practice in traditional Chinese medicine. As one of the combination drugs for the treatment of hypertension (patent number: CN201810790515.4), ET exhibited good efficacy against age-related hypertension in previous studies [13, 14].

In this study, we investigated the effects of ET on the intestinal microbiota and its metabolites. We sequenced the 16S rRNA V4 region in the intestinal microbiota and performed gas chromatography-mass spectrometry (GC-MS) on fecal metabolites and integratively analyzed their correlation to explore the potential mechanism underlying the antihypertensive effects of ET.

## 2. Methods

### 2.1. Preparation of ET

Formula particles of *Eucommia ulmoides* and *Tribulus terrestris* (Tianjiang Phar, *Eucommia ulmoides* formula granules: No. 1609169, 1 g/package; *Tribulus terrestris* formula granules: No. 1502063, 0.5 g/package, Jiangyin, China) were purchased from the Affiliated Hospital of Shandong University of Traditional Chinese Medicine. The formula particles were dissolved with ultrapure water to a concentration of 1.63 g raw herbs/ml (*i.e.*, 0.81 g *Eucommia* *ulmoides* + 0.81 g *Tribulus* *terrestris*/ml).

### 2.2. In Vivo Drug Administration

The study was approved by the Faculty of Medicine and Health Sciences Ethics Committee for Animal Research (*No.* AF/SC-08/02.0), Affiliated Hospital of Shandong University of Traditional Chinese Medicine (Jinan, China). All efforts were made to alleviate the pain of experimental animals. All animals and experimental procedures in this study were performed in keeping with the Regulations on the Management of Experimental Animals of People's Republic of China (revised version March 1, 2017). Fourteen male spontaneously hypertensive rats (SHRs, 18 months old) and 7 WKY rats of the same age were purchased from Beijing Vital River Laboratory Animal Technology Co., Ltd. (certificate: SCXK (Jing) 2016-0011). The rats were maintained under the conditions of constant temperature (20-22°C), constant humidity (50-60%), and fixed light and dark alternation (12 h/12 h). All rats had access to Cobalt 60-sterilized fodder (Vital River Laboratory Animal Technology Co., Ltd., Beijing, China) and ultrapure water *ad libitum*. After a 5-day adaptive feeding, SHRs were randomly divided into the ET group and the SHR group. SHRs in the ET group were given 1.0 g/kg/d ET suspension (*i.e.*, 0.5 g *Eucommia* *ulmoides* + 0.5 g *Tribulus* *terrestris*/*k*g/d) intragastrically, which was equivalent to 0.5 g/kg/d *Eucommia* *ulmoides* + 0.5 g/kg/d *Tribulus* *terrestris* in human clinical application, for 6 weeks. The WKY rats and SHRs were administered the same volume of normal saline.

### 2.3. Blood Pressure Measurement and Sample Collection

Body weight was recorded weekly. Blood pressure was measured weekly in conscious rats by a tail-cuff method (MRBP-10, IITC Life Science, USA). Rats were allowed to adapt to the pressure measurement environment for 1 week before formal recording. Rats were placed in the ALC-HTP animal system in quiet and awake states. The systolic pressure and heart rate of the rats were measured. The average of 5 measurements was recorded in parallel with an interval of 1 min. Stool samples were collected by pinching the tail to let rats defecate under stress and collected from the anus to a sterile centrifuge tube directly. All samples were stored at -80°C.

### 2.4. HE Staining

The colons were removed on ice and fixed in neutral paraformaldehyde for 48 h. The colon tissues were embedded in paraffin, sectioned at a thickness of 4 *μ*m, stained with hematoxylin-eosin (HE), and sealed with neutral balsam. The slides were observed and photographed under a light microscope.

### 2.5. Intestinal Microbiota Analysis

Sequencing of 16S rRNA was performed by Novogene Technology Co., Ltd. (Beijing, China, Project No. p101sc17020776-01-s1-3-2).

#### 2.5.1. DNA Extraction and PCR Amplification

DNA concentration and purity were determined on 1% agarose gels. The DNA concentration was adjusted to 1 ng/*μ*l. The V4 region within the 16S rRNA gene was amplified using specific primers. The primer sequences were 5′-GTGCCAGCMGCCGCGGTAA-3′/5′-GGACTACHVGGGTWTCTAAT-3′. PCR products were confirmed with 2% agarose gel electrophoretic analysis, and samples with bright and distinct bands between 400 and 450 bp were extracted and purified with a Qiagen Gel Extraction Kit (Qiagen, Germany).

#### 2.5.2. Library Construction and Sequencing

Sequencing libraries were generated using the TruSeq® DNA PCR-Free Sample Preparation Kit (Illumina, USA) following the manufacturer's recommendations. The library quality was assessed on the Qubit@ 2.0 Fluorometer (Thermo Scientific, United States) and Agilent Bioanalyzer 2100 system. Finally, the library was sequenced on an Illumina HiSeq 2500 platform, and 250 bp paired-end reads were generated.

#### 2.5.3. Data Processing and Analysis

Paired-end reads were assigned to samples based on their unique barcode and were truncated by cutting off the barcode and primer sequence. For sequence assembly, paired-end reads were merged using FLASH (V1.2.7). High-quality clean tags were utilized according to the QIIME (V1.7.0) quality-controlled process. The tags were compared with the reference database (Gold database) using the UCHIME algorithm (UCHIME Algorithm) to detect and remove chimeric sequences. Next, the effective tags were obtained. Sequence analysis was performed by Uparse software (Uparse v7.0.1001). Sequences with ≥97% similarity were assigned to the same OTUs. For each representative sequence, Green Gene Database 3 was used based on the RDP classifier (version 2.2) algorithm to annotate taxonomic information. Multiple sequence alignment was performed using MUSCLE software (version 3.8.31).

Alpha diversity was analyzed by PD-whole tree and observed species. Beta diversity was manifested as PCoA and NMDS analysis. LEfSe analysis was employed to compare species with significant differences among groups. The Spearman correlation coefficient was calculated to determine the potential correlation between the intestinal microbiota and metabolites.

### 2.6. Fecal Metabolite Gas Chromatography-Mass Spectrometry (GC-MS) Analysis

The GC-MS assay was performed by OE Biotech Co., Ltd. (Shanghai, China, Project No. QDOE2017s907).

#### 2.6.1. GC Conditions

A DB-5MS fused-silica capillary column (30 m × 0.25 mm × 0.25 *μ*m, Agilent J&W Scientific, Folsom, CA, USA) was utilized to separate the derivatives at a constant flow of 1 ml/min helium. One microliter of sample was injected in split mode at a 20 : 1 split ratio by the autosampler. The injection temperature was 280°C, the interface was set to 150°C, and the ion source was adjusted to 230°C. The temperature rise program was followed by an initial temperature of 60°C for 2 min, a 10°C/min rate up to 300°C, and a temperature of 300°C for 5 min.

#### 2.6.2. MS Conditions

Mass spectrometry was performed using the full-scan method with a range of 35 to 750 (*m*/*z*).

#### 2.6.3. Data Processing and Analysis

Base File Converter software was used to convert the raw data to.abf format, and then, the.abf data were imported into the MD-DIAL software for data processing. Metabolites were annotated using the LUG database (untargeted database of GC-MS from Lumingbio). Partial least square analysis (PLS-DA) and orthogonal partial least square analysis (OPLS-DA) were utilized to distinguish the overall differences in metabolic profiles among groups and to identify the different metabolites. Student's *t*-test and fold change analysis were employed to compare the different metabolites between the two groups, where metabolites with VIP values larger than 1.0 and *p* values less than 0.05 were considered to be differential metabolites. The KEGG database was employed to analyze metabolic pathway enrichment.

### 2.7. Statistical Analysis

Statistical analyses were performed using SPSS 20.0. The values are presented as the means ± standard deviation. Unless otherwise stated, statistical comparisons were performed using a single-factor analysis of variance (ANOVA) followed by the LSD test. In this study, *p* values less than 0.05 were considered to be significantly different. The Pearson correlation coefficient was utilized in the correlation analysis between the intestinal microbiota and fecal metabolites.

## 3. Results

### 3.1. Blood Pressure and Morphology of Colon Tissues

**Figure 1:**
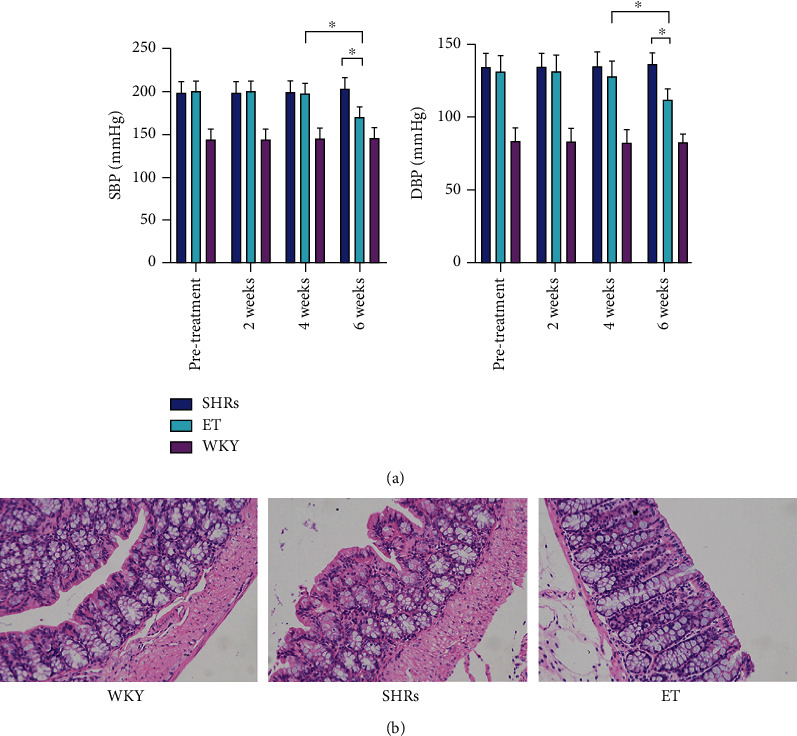
Blood pressure and colon tissue morphology. (a) Systolic and diastolic blood pressure levels after 6 weeks of ET administration (mmHg). ^∗^*p* < 0.05*vs*. WKY rats. SBP: systolic blood pressure; DBP: diastolic blood pressure. (b) Morphological changes in HE-stained colon tissues after 6 weeks of ET administration (200x).

Before treatment, the systolic and diastolic blood pressure of the ET group was not significantly different from that of the SHR group (*p* > 0.05). After 6 weeks of treatment, both systolic and diastolic blood pressures in the ET group were significantly lower than those in the SHR group (*p* < 0.05) (Figure 1(a)). As shown in Figure 1(b), intestinal mucosal injury was significantly more severe in the SHR group than in the WKY group, whereas ET treatment clearly improved this pathological manifestation of colon tissues in SHRs.

### 3.2. ET Changes the Diversity of Intestinal Microbiota in SHR Rats

**Figure 2:**
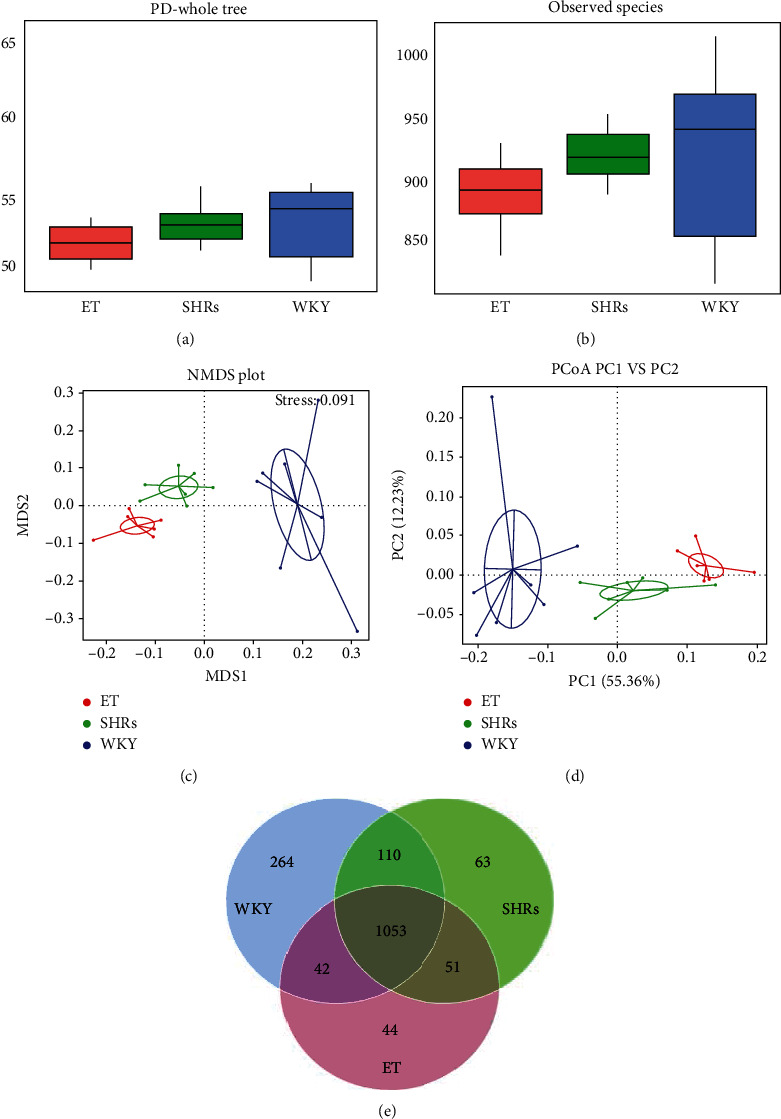
*α*- and *β*-diversity of intestinal microbiota. (a and b) PD-whole tree and observed species analysis. The black line in the middle of the rectangle in every group represents the median. (c and d) The more similar the composition of different samples in the PCoA analysis and NMDS analysis plot is, the closer the position in the plot is. The plot indicates that there is a significant community difference among the three groups. (e) The Venn diagram shows the common and different intestinal microbiota of the three groups.

We employed *α*-diversity and *β*-diversity to describe the intra- and intergroup differences of microbiota. The results of PD-whole tree and observed species analysis (*α*-diversity) showed that both the abundance and diversity of the intestinal microbiota among the three groups could be ranked as follows: WKY > ET > SHRs (Figures 2(a) and 2(b)).

*β*-Diversity was described by a linear model (PCoA analysis) and a nonlinear model (NMDS analysis). Both PCoA analysis and NMDS analysis showed that there were different clusters among the three groups, suggesting significant community differences (Figures 2(c) and 2(d)).

Overall, the SHR, WKY and ET groups shared 1053 common species, while the ET group had 44 unique species and the SHR group had 63 unique species (Figure 2(e)).

### 3.3. Screening of Potential Intestinal Microbiota Biomarkers

**Figure 3:**
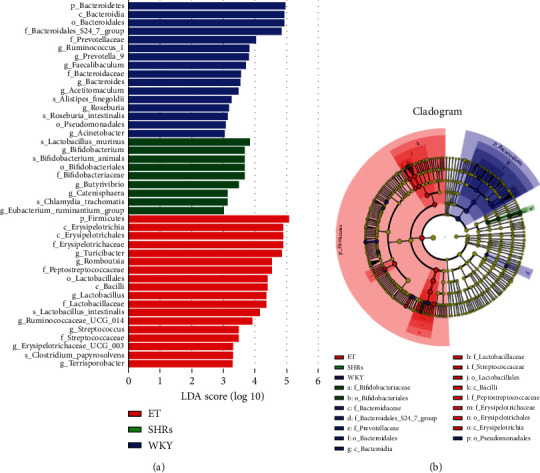
Potential biomarkers, as determined by LEfSe analysis. (a) Linear discriminant analysis value distribution histogram. The prefixes of p, c, o, f, g, and s before the species represent six different taxonomic levels: phylum, class, order, family, genus, and species. (b) Evolutionary branch diagram. Each small circle indicates a classification at this level, and the size of the circle was proportional to the relative abundance. Coloring principle: biomarkers of different species colored following the corresponding group. Yellow stands for species with no significant difference, while red nodes and green nodes represent the key microbial communities that play important roles in their respective groups.

We conducted LEfSe analysis to screen the potential biomarkers of groups. The results showed that the potential biomarkers of the WKY group were *Ruminococcus-1*, *Prevotella-9*, *Faecalibaculum*, *Bacteroides*, *Acetitomaculum*, *Roseburia*, and *Acinetobacter*. The potential biomarkers of the SHR group were *Bifidobacterium*, *Butyrivibrio*, *Catenisphaera*, and *Eubacterium_ruminantium_group*. *Turicibacter*, *Romboutsia*, *Lactobacillus*, *Ruminococcaceae_UCG_014*, *Streptococcus*, *Erysipelotrichaceae_UCG_003*, and *Terrisporobacter* were the potential biomarkers of the ET group (Figure 3).

### 3.4. Identification of Metabolites

**Figure 4:**
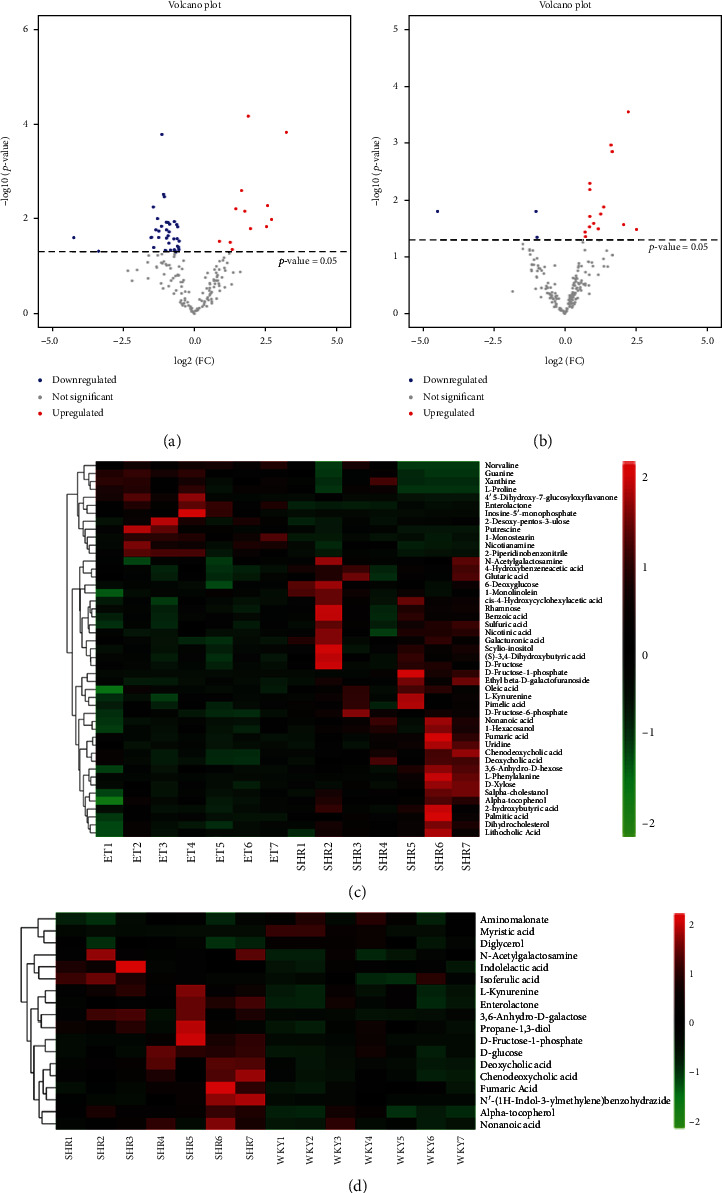
Identification of fecal metabolites. (a and b) Volcano plot of differential metabolites. Red represents upregulated metabolites, and blue represents downregulated metabolites. (c and d) Heatmap of differential metabolites. The horizontal direction is the name of fecal samples, while the longitudinal direction represents the clustering of metabolites; the shorter the clustering branches are, the more similar the color is, and the higher the similarity is.

We screened differential metabolites by determining VIP > 1, FC > 2.0, or FC < 0.5 and *p* values < 0.05 (Figures 4(a) and 4(b)). Ultimately, 8 potential biomarkers were identified that may be associated with reduced blood pressure (Table 1). A heat map of differential metabolites suggested that the metabolic patterns and metabolic function of WKY rats and SHRs were different and that ET treatment affected them (Figures 4(c) and 4(d)).

#### 3.4.1. Metabolic Pathway Analysis

**Figure 5:**
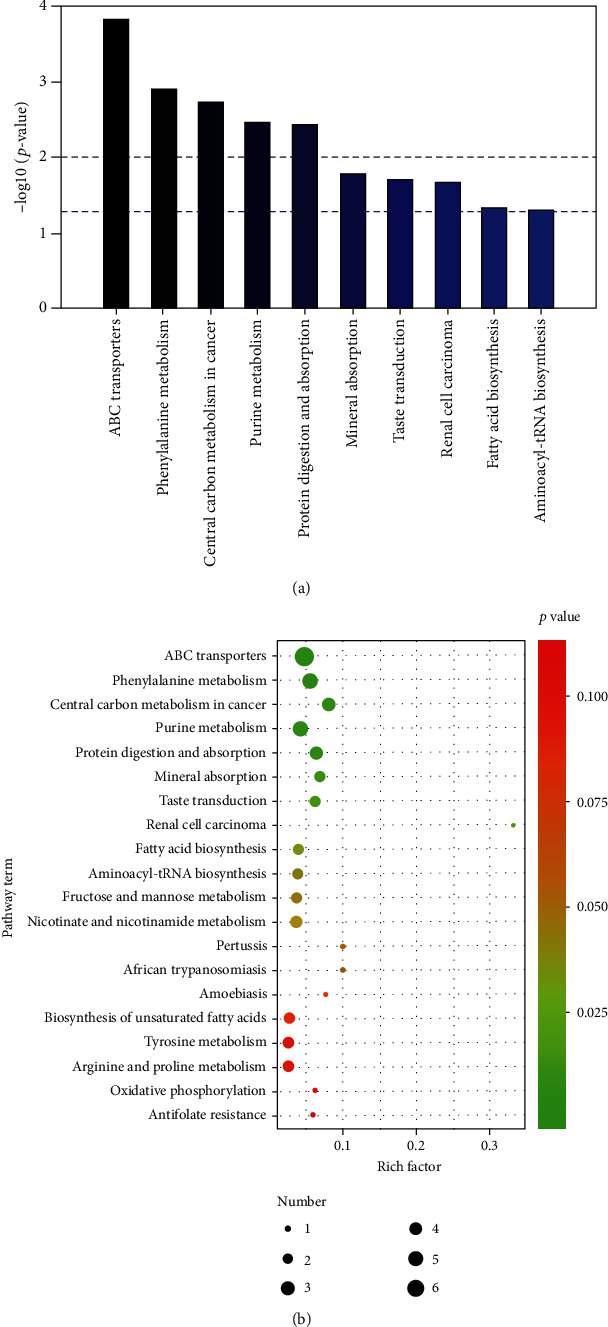
Metabolic pathway analysis of potential biomarkers. Ten metabolic pathways are shown in (a). The number of metabolites involved in metabolic pathways and the *p* values are shown in (b). The smaller the *p* value is, the more significant the enrichment is.

To explore the metabolic function of the metabolites, we ran the Metaboanalyst pathway analysis module with Metaboanalyst software. The results of this analysis showed that ABC transporters, phenylalanine metabolism, central carbon metabolism in cancer, purine metabolism, protein digestion and absorption, mineral absorption, taste transduction, renal cell carcinoma, fatty acid biosynthesis, and aminoacyl-tRNA biosynthesis were the top 10 enrichment and topological pathways (Figure 5).

### 3.5. Correlation between Potential Biomarkers and Different Microbiota

The correlation of potential biomarkers of metabolomics and intestinal microbiota was analyzed by the Spearman correlation coefficient, since intestinal microbiota regulate the metabolic function of the host [15].

*Acinetobacter* had a positive correlation with the metabolic level of (s)-3,4-dihydroxybutyric acid, 2-hydroxybutyric acid, 6-deoxyglucose, chenodeoxycholic acid, deoxycholic acid, D-fructose, and D-fructose-6-phosphate and a negative correlation with 1-monostearin, 2-desoxy-pentos-3-ulose, 2-piperideinobenzonitrile, and 4′,5-dihydroxy-7-glucosyloxyflavanone.

*Erysipelotrichaceae* had a positive correlation with the metabolic level of 1-monostearin and 4′,5-dihydroxy-7-glucosyloxyflavanone and a negative correlation with 1-hexacosanol, 3,6-anhydro-D-hexose, 5-alpha-cholestanol, alpha-tocopherol, chenodeoxycholic acid, deoxycholic acid, and D-fructose-1-phosphate and D-fructose-6-phosphate.

**Figure 6:**
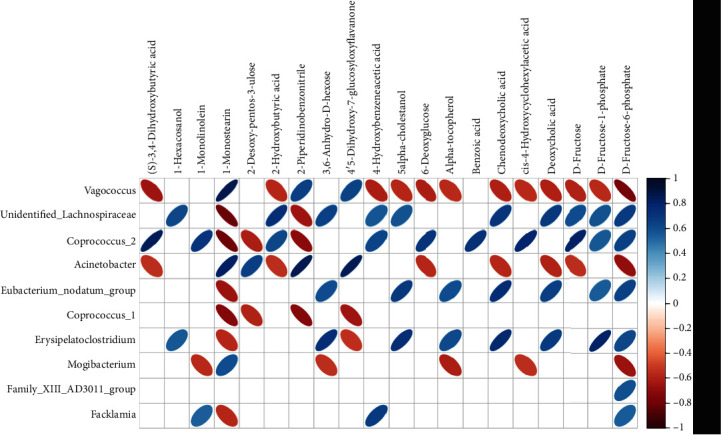
Correlation of intestinal microbiota and metabonomics. The vertical direction represents the differential bacteria at the level of 16S OTUs (species, genus), and the horizontal direction represents the names of differential metabolite. The value range of the correlation coefficient is (-1, +1). The intestinal microbiota and fecal metabolites show ∣rho | > = 0.7 and *p* < 0.05, which is considered to demonstrate a high correlation and a significant effect. The red oval indicates a positive correlation, and the blue oval indicates a negative correlation between the intestinal microbiota and fecal metabolites.

*Unidentified_Lachnospiraceae* had a positive correlation with the metabolic level of 1-monostearin and 2-piperidinobenzonitrile and a negative correlation with 1-hexacosanol, 2-hydroxybutyric acid, 3,6-anhydro-D-hexose, 4-hydroxybenzeneacetic acid, 5-alpha-cholestanol, chenodeoxycholic acid, deoxycholic acid, D-fructose, D-fructose-1-phosphate, and D-fructose-6-phosphate (Figure 6).

## 4. Discussion

ET administration demonstrated beneficial effects on blood pressure and increased the diversity of the intestinal microbiota and intestinal mucosal integrity. Correlation analysis suggested that the intestinal microbiota regulated greater quantities of metabolites in certain metabolic pathways, thereby reducing blood pressure.

Several reports supported the prediction potential of the intestinal microbiota in hypertension [16]. The pathological process of hypertension clearly damages the diversity of intestinal microbiota, while imbalances in the intestinal microbiota contribute to the development of hypertension [17]. In our study, the diversity of the intestinal microbiota in WKY rats was significantly higher than that in SHR rats. After ET administration, the intestinal microbiota diversity of SHRs clearly improved, which was beneficial for SHRs. However, we noticed that ET administration did not restore the diversity of the intestinal microbiota to normal levels, which may be attributable to the long-lasting pathological process of hypertension.

*Butyrivibrio* is an important butyric acid-producing genus. Damage to the colonic epithelium is widespread under the condition of hypertension [18], while butyric acid is an important fuel for repairing the colonic epithelium. A significant change in *Streptococcus* abundance is observed between normotensive populations and people taking antihypertensive drugs in clinical studies, which suggests that *Streptococcus* responds to changes in blood pressure (even after blood pressure control) [19]. *Lactobacillus* is a microorganism to which antihypertensive effects have been attributed [20]. In the present study, the relative abundances of *Streptococcus* and *Lactobacillus* were significantly increased after ET administration, which supports the efficacy of ET in lowering blood pressure.

Treatment with *α*-tocopherol at a moderate level has been indicated to have an anti-inflammatory effect. However, treatment with excessive quantities of *α*-tocopherol increased the risk of mortality in humans according to a meta-analysis [21]. A study also showed that alpha-tocopherol affected the level of renal (Na^+^/K^+^) ATPase in a dose-dependent manner, and the altered Na+/K+ ratio is related to hypertension [21]. Chenodeoxycholic acid was considered a natural ligand of the farnesoid X receptor in alleviating elevated blood pressure in spontaneously hypertensive rats [22]. The activities of 7*α*-dehydroxylated enzyme and 7*β*-dehydroxylated enzyme, which both participate in the process of bile acid conversion to deoxycholic acid and other secondary metabolites, are completely involved in the intestinal microbiota [23]. Notably, the related metabolism and transformation of chenodeoxycholic acid and deoxycholic acid in bile metabolism were driven by the intestinal microbiota. Kynurenine, a tryptophan metabolite in the gastrointestinal tract, was significantly increased in SHRs or in hypertensive patients [24]. Kynurenine is derived from the indoleamine 2,3-dioxygenase pathway, which is regulated by the intestinal microbiota [25]. Enterolactone was the metabolite of linseed lignan, which was determined to have the ability to favorably modulate SBP in older adults, which would help to decrease risk in cardiovascular disease [26]. Interestingly, enterolactone is one of the substances that distinguishes the metabolism of different intestinal microbial models [27]. In the present study, *α*-tocopherol, chenodeoxycholic acid, and kynurenine were downregulated after ET administration, while enterolactone was upregulated. Other metabolites, such as deoxycholic acid, fumaric acid, N-acetylgalactosamine, and nonanoic acid, were also downregulated. The mechanism governing the involvement of these metabolites in blood pressure regulation has not been elucidated. Remarkably, changes in those metabolites were at least partly regulated by the intestinal microbiota [28, 29]. It is known that the intestinal microbiota contributes to inflammatory mechanisms, neurobiological functions, and immune responses [24].

Our fecal metabonomics data suggested that metabolites affected by ET were primarily enriched in ABC transporters, phenylalanine metabolism, central carbon metabolism in cancer, purine metabolism, L-tyrosine digestion and absorption, mineral absorption, taste transduction, renal cell carcinoma, fatty acid biosynthesis, and aminoacyl-tRNA biosynthesis.

### 4.1. Protein-, Amino Acid-, and Purine-Related Metabolism

ABC transporters contribute primarily to energy metabolism and transport various substrates, including ions, peptides, lipids, and proteins, involving many key physiological and pathological processes. Although these transporters have not been related to hypertension, they are closely related to atherosclerosis, gout and hyperuricemia, coronary heart disease, and other diseases that are high-risk factors for hypertension [24, 30, 31].

It is worth noting that phenylalanine metabolism is a precursor to catecholamine biosynthesis. There is an abnormal state of phenylalanine metabolism in spontaneously hypertensive rats. The intake of aromatic amino acids, including phenylalanine (AAAs), increases the risk of hypertension according to a clinical study of 4288 individuals [32]. In our study, ET treatment significantly downregulated the phenylalanine-related metabolic pathway, which we hypothesized is one of the mechanisms by which ET exerts its hypotensive effects.

Abnormal purine metabolism is an independent risk factor for hypertension. Hyperuricemia is caused by abnormal purine metabolism, which leads to a decrease in nitric oxide synthesis, changes in the biological behavior of vascular smooth muscle cells, inflammation, oxidative stress, and an increased risk of hypertension. ET treatment effectively downregulated purine metabolism levels.

### 4.2. Lipid- and Fatty Acid-Related Metabolism

Fatty acid metabolism has multiple effects on the development of hypertension. Short-chain fatty acids are among the primary products of intestinal microbial metabolism, and they affect the blood pressure level of the host through Gpr41. Acute short-chain fatty-acid administration can decrease the blood pressure levels of spontaneously hypertensive rats [33], improve nutrient digestibility, inhibit the proliferation of harmful bacteria, and increase the abundance of beneficial bacteria [34]. The intestinal microbiota are the only source of short-chain fatty acids in the host. In this study, ET treatment improved the function of the intestinal microbiota, thereby affecting the metabolism of fatty acids, which helped to maintain overall health.

### 4.3. Cancer-Related Metabolism

There is a definite relationship between the pathogenesis of hypertension and cancer. These diseases share similar risk factors and pathophysiology mechanisms. Hypertension increased the risk of mortality from cancer, particularly renal cell carcinoma, according to a retrospective study conducted in 2002 [35]. In the present study, after the 6-week administration of ET, both central carbon metabolism in cancer and renal cell carcinoma-related levels were changed in SHRs. Interestingly, both *Eucommia ulmoides* and *Tribulus terrestris* are considered to be kidney tonifying agents in traditional Chinese medicine.

### 4.4. Mineral Absorption

Minerals are necessary elements to maintain physiological activities. It is known that the levels of certain elements, such as Ni and Co, in the serum are negatively correlated with systolic and diastolic blood pressure, while the serum Se level is positively correlated with blood pressure. Physical function declines with aging, and disorders of mineral absorption and metabolism are exacerbated, which is not conducive to the regulation of blood pressure. In this study, the body's absorption of minerals was significantly improved after ET administration, which was a notable example of the multitarget effect of traditional Chinese medicine.

### 4.5. Taste Transduction

Taste transduction is related to neuroendocrine regulation, and the mechanisms of sensing taste in the oral cavity have been thoroughly characterized. Several studies [36] have demonstrated the existence of ingested substances along the gastrointestinal tract as additional mechanisms, showing that the recognition receptor of ingested substances released gastrointestinal hormones and regulated energy homeostasis through the vagus nerve and enteric afferents. The taste conduction pathway was improved by ET, indicating that ET may have a corresponding effect on neuroendocrine regulation.

The correlations between *α*-tocopherol, chenodeoxycholic acid, and deoxycholic acid were highly closely correlated with the abundance of *Eubacterium* according to the Spearman correlation analysis. A clinical study also showed that the number of *Eubacterium rectale* bacteria was increased in the intestines of patients with hypertension [37]. Our study also detected decreased levels of *Eubacterium* after the administration of ET. This microbe may be one of the target microbiota of ET that play important roles in lowering blood pressure. The effects of *Erysipelotrichaceae* on the host still warrant further research; however, considerable evidence [38] indicates that *Erysipelotrichaceae* is related to lipid metabolism, which is consistent with our results. In addition, *Erysipelotrichaceae* in SHRs also exhibited a correlation with flavonoid metabolism. It is known that flavonoids are important pharmacophore monomers in both *Eucommia ulmoides* and *Tribulus terrestris* [39]. Flavonoids have a significant protective effect on the cardiovascular system, as well as pharmacological activities of lowering blood pressure and blood lipids, reducing oxidation and inhibiting inflammation [40]. Because *Erysipelotrichaceae* was identified as a biomarker of ET in this study, we speculated that ET may regulate blood pressure by targeting lipid and flavonoid metabolism by increasing the abundance of *Erysipelotrichaceae*. Meanwhile, an increase in the abundance of *Erysipelotrichaceae* after ET treatment helped to regulate the structure of the intestinal microbiota of SHRs. We determined that ET treatment affected metabolism and enhanced the utilization of flavonoids in SHRs.

## 5. Conclusion

The combination of *Eucommia ulmoides* and *Tribulus terrestris* yielded a good antihypertensive effect, and this efficacy was related to the improvement of the intestinal microbiota and their related metabolites. We hope that these results serve to elucidate the antihypertensive effects of the combination of *Eucommia ulmoides* and *Tribulus terrestris*.

## Figures and Tables

**Table 1:** Fecal metabonomics.

Biomarker	HMDB	KEGG	Variation trend (SHRs : WKY^a^/ET : SHRs^b^)
Alpha-tocopherol	HMDB0001893	C02477	↑↓
Chenodeoxycholic acid	HMDB0000518	C02528	↑↓
Deoxycholic acid	HMDB0000518	C02528	↑↓
Enterolactone	HMDB0006101	C18165	↓↑
Fumaric acid	HMDB0000134	C00122	↑↓
L-Kynurenine	HMDB0000684	C00328	↑↓
N-Acetylgalactosamine	HMDB0000212	C01074	↑↓
Nonanoic acid	HMDB0000847	C01601	↑↓

^a^Trends of the SHR group compared with the WKY group. ^b^Trends of the ET group compared with the SHR group. ↑: upregulated; ↓: downregulated.

## Data Availability

The datasets used and/or analyzed during the present study are available from the corresponding author on reasonable request.
